# Silent sinus syndrome after rhinoplasty: a case report

**DOI:** 10.1080/23320885.2020.1788947

**Published:** 2020-07-14

**Authors:** Mert Ersan, Arda Özdemir, Serdar Mehmet Gültan

**Affiliations:** Department of Plastic Reconstructive and Aesthetic Surgery, Ankara University School of Medicine, Ankara, Turkey

**Keywords:** Enophthalmos, paranasal sinuses, rhinoplasty, Silent Sinus Syndrome

## Abstract

Silent Sinus Syndrome is a rare syndrome that usually involves the maxillary sinus. In this case report, we present a rare case diagnosed as Silent Sinus Syndrome with frontal, ethmoid and maxillary sinus involvement which was presented with periorbital complaints after the rhinoplasty operation performed in our clinic.

## Introduction

Although Silent Sinus Syndrome was described in two asymptomatic patients by Montgomery in 1964, the definition of these symptoms as a syndrome was made by Soparkar in 1994 [1,2]. As a result of obstruction of the osteomeatal complex, the maxillary sinus is hypoventilated and negative pressure is created within the sinus due to the gradual diffusion of intra-sinus gases into the capillary circulation [3]. The negative pressure arises from mucus and other secretions that progressively accumulate in the sinus and causes a gradual collapse in the orbital floor within weeks or months [3]. However, as it can be understood from the name of the syndrome, although the majority of patients have radiological sinus wall retraction and total or near-total opacification of the sinus; sinusitis and similar sinus pathologies are not observed [4,5]. Although the maxillary sinus is mostly affected in Silent Sinus Syndrome, other paranasal sinuses, including the frontal and ethmoid sinus, can be affected individually or combined fashion [2]. Silent Sinus Syndrome after rhinoplasty was first introduced by Eloy et al. in a patient with isolated maxillary sinus involvement. Regarding Silent Sinus Syndrome after rhinoplasty, to our knowledge, this case report is the second case in the literature; however it is the first case in which three paranasal sinuses are affected together [5].

## Case report

**Figure 1.**
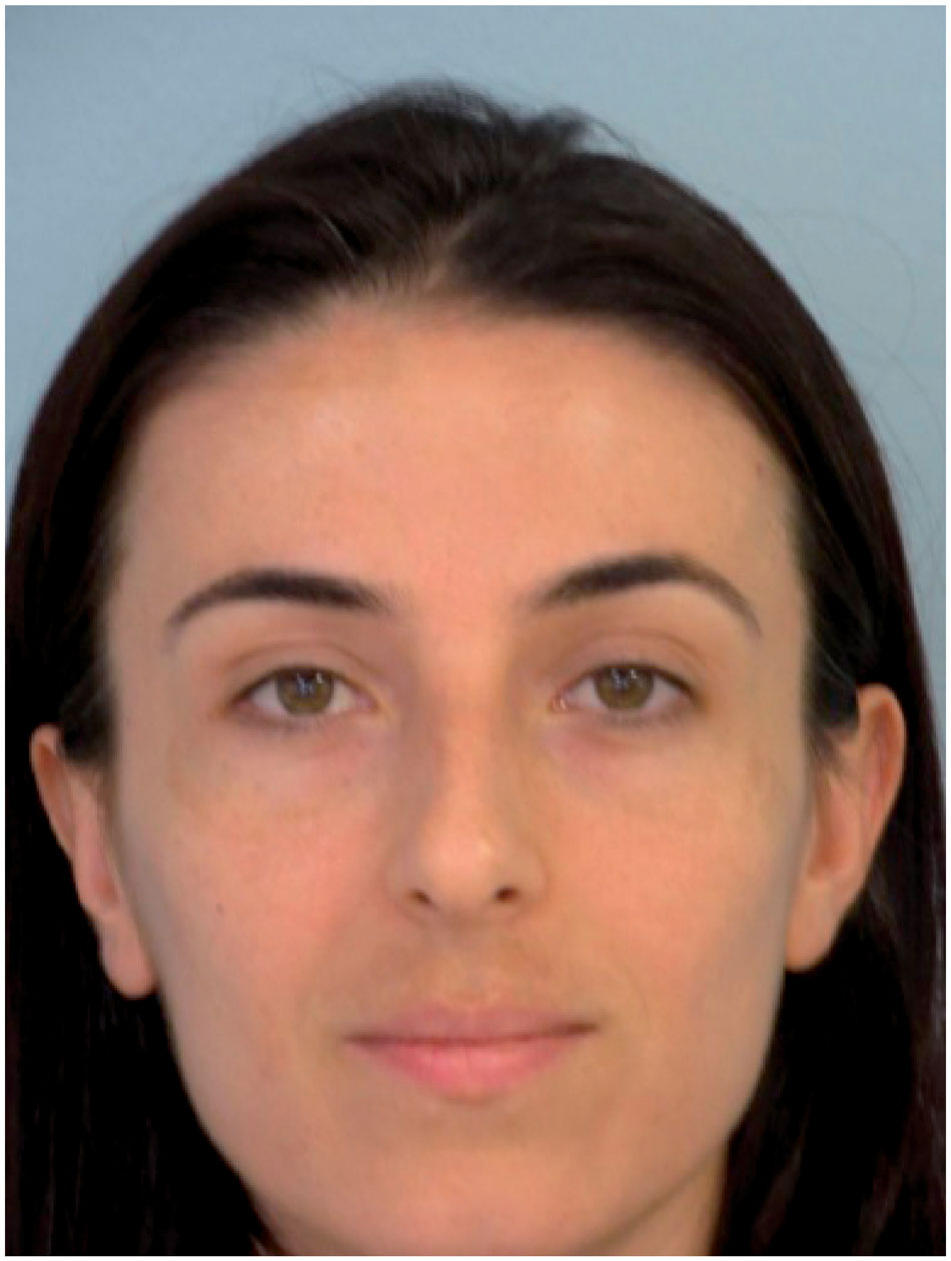
Preoperative frontal view of the patient.

**Figure 2.**
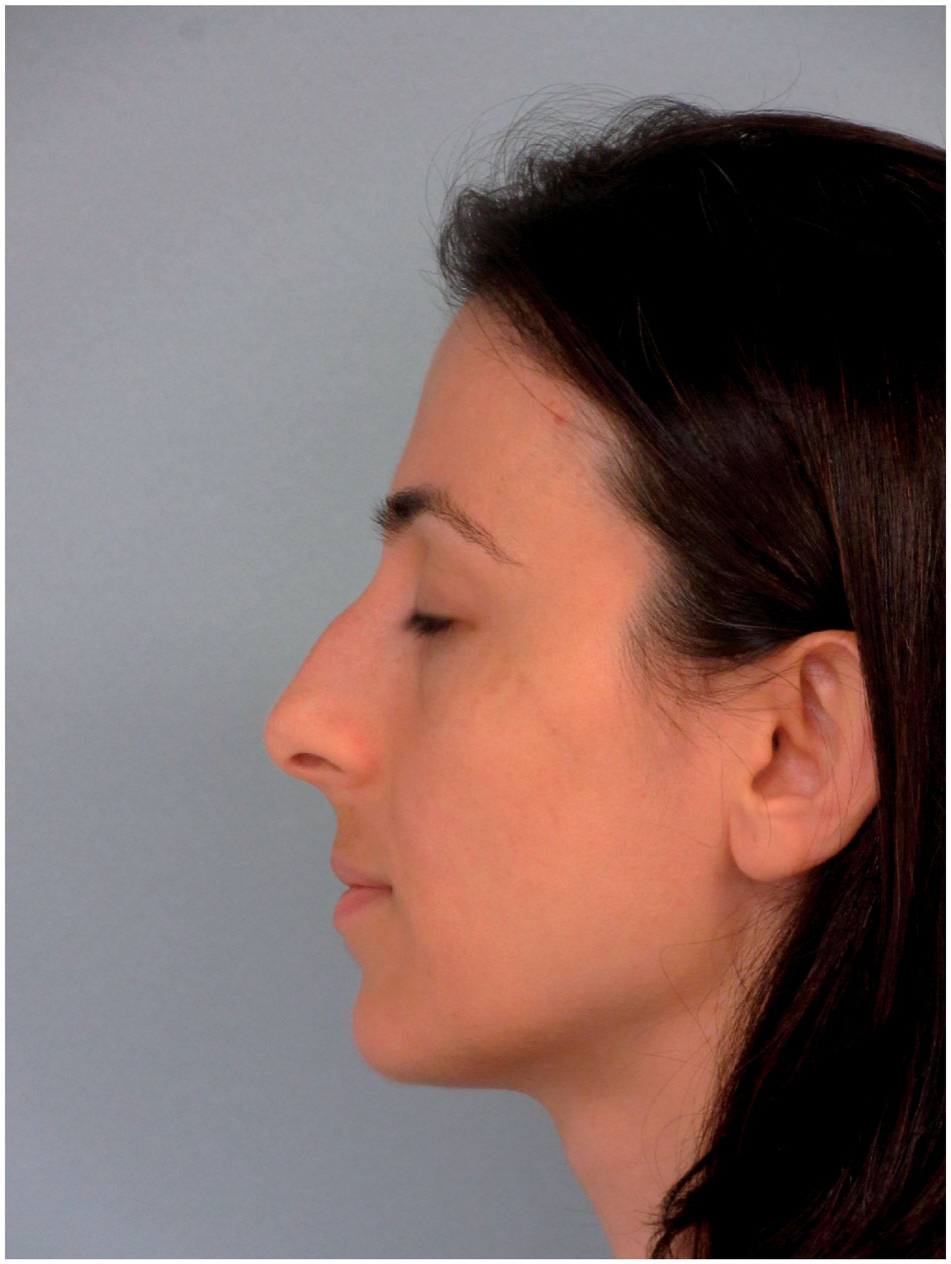
Preoperative lateral view of the patient.

**Figure 3.**
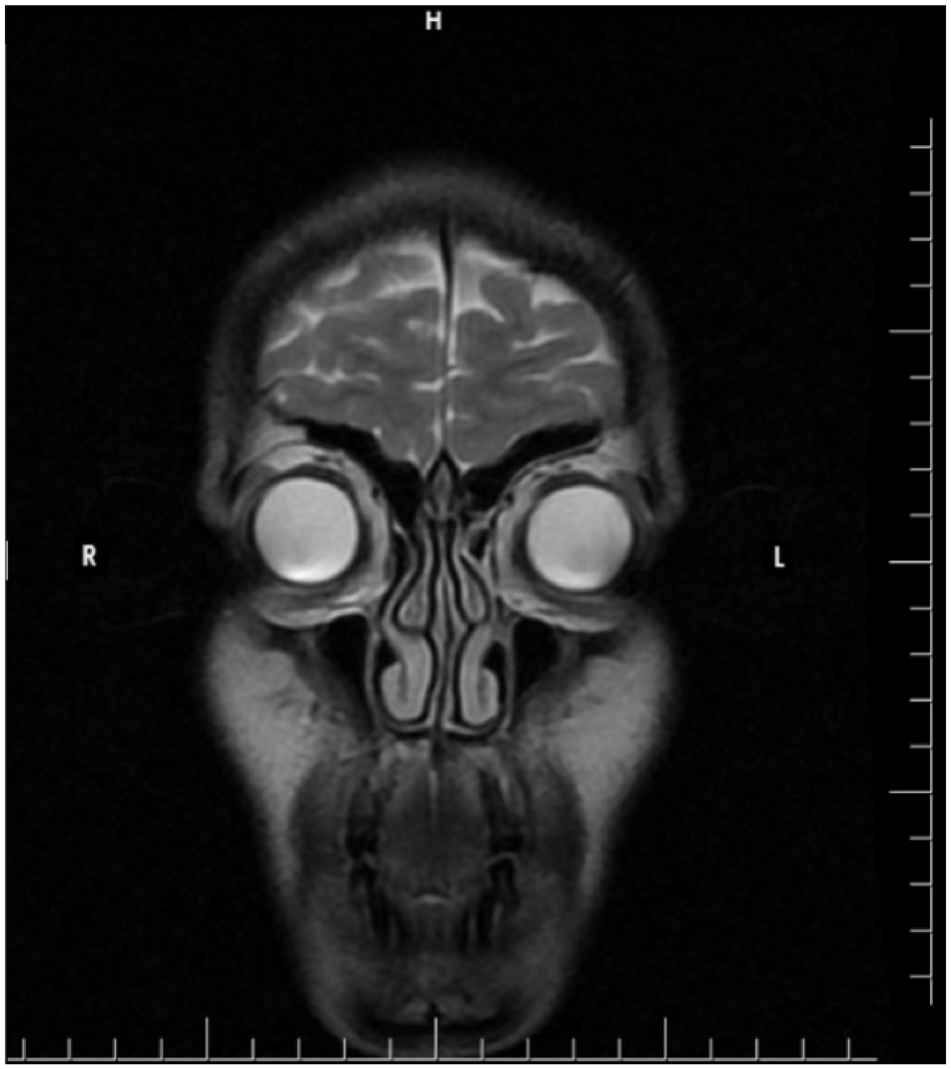
The MR image of the patient taken before the application. MR imaging shows that there are no pathology in bilateral frontal, ethmoid and maxillary sinuses.

A 32-year-old woman presented to our clinic with aesthetic concerns about her nose. The history of the patient revealed that she had no additional disease, no active drug use or allergy and no previous intervention to the facial area. On physical examination of the nose, the dorsal hump was inspected. Nasal speculum examination revealed that the bilateral airway was sufficiently open. The patient was prepared for rhinoplasty operation without additional radiological examination since the patient’s Cranial MR, which was recently taken for the headaches, showed no abnormalities for nasal airways and the patient had no problem with breathing. The preoperative photos taken during the patient’s initial application and MR images are shown in Figures 1–3.

The patient was prepared for rhinoplasty operation under general anesthesia. Complete blood count, renal function tests, liver function tests, glucose and HbA1c levels, coagulation parameters and viral markers were within the normal range.

Open structural rhinoplasty was initiated with the elevation of the nasal flap with transcolumellar and infracartilaginous incisions. Cartilage excisions were performed in a way that the L shaped septum preserved with the safety margins of 1 cm caudally and cephalically. During the operation low to high lateral osteotomies were performed following the paramedian osteotomies and the nasal roof was closed. As a part of the operation, the inferior conchas were lateralized. The operation was terminated following the internal and external nasal splint applications. No abnormality was encountered intraoperatively.

**Figure 4.**
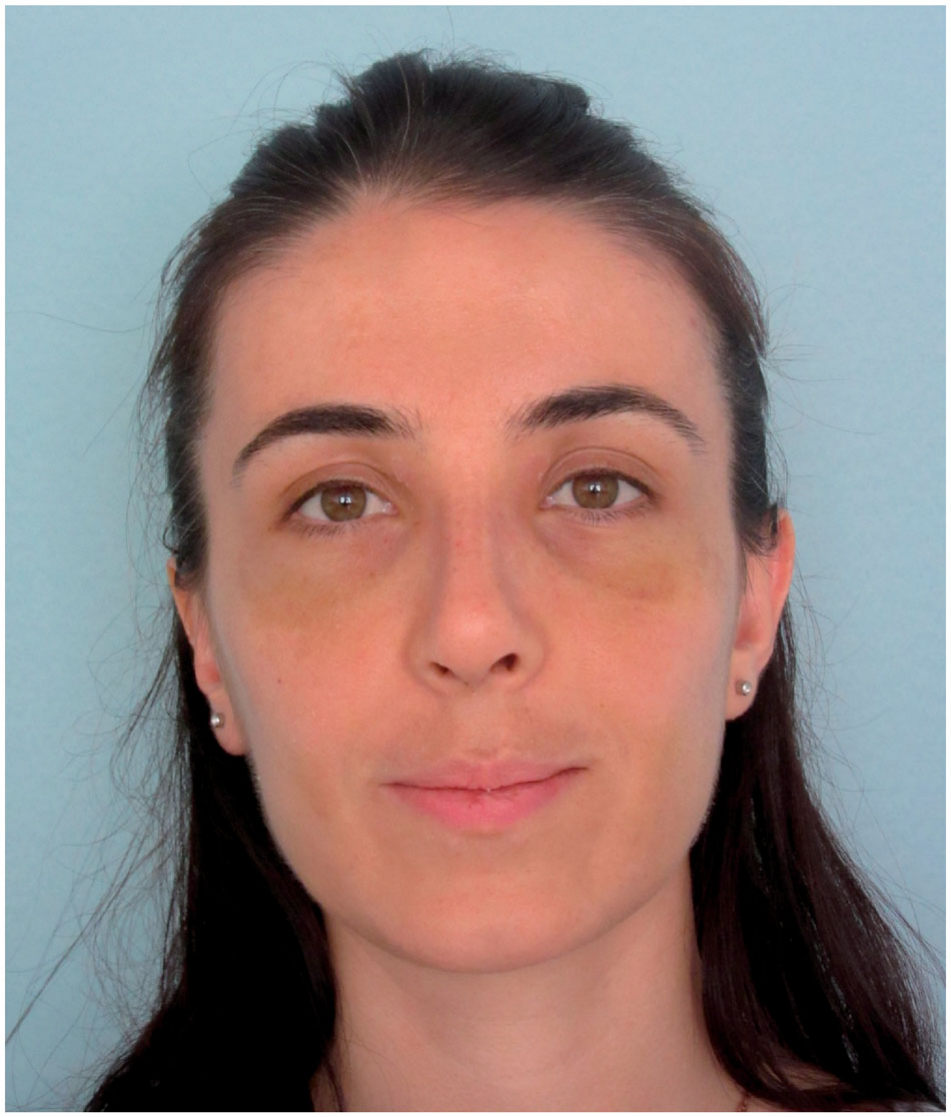
Frontal view of the patient on the 8th postoperative day.

**Figure 5.**
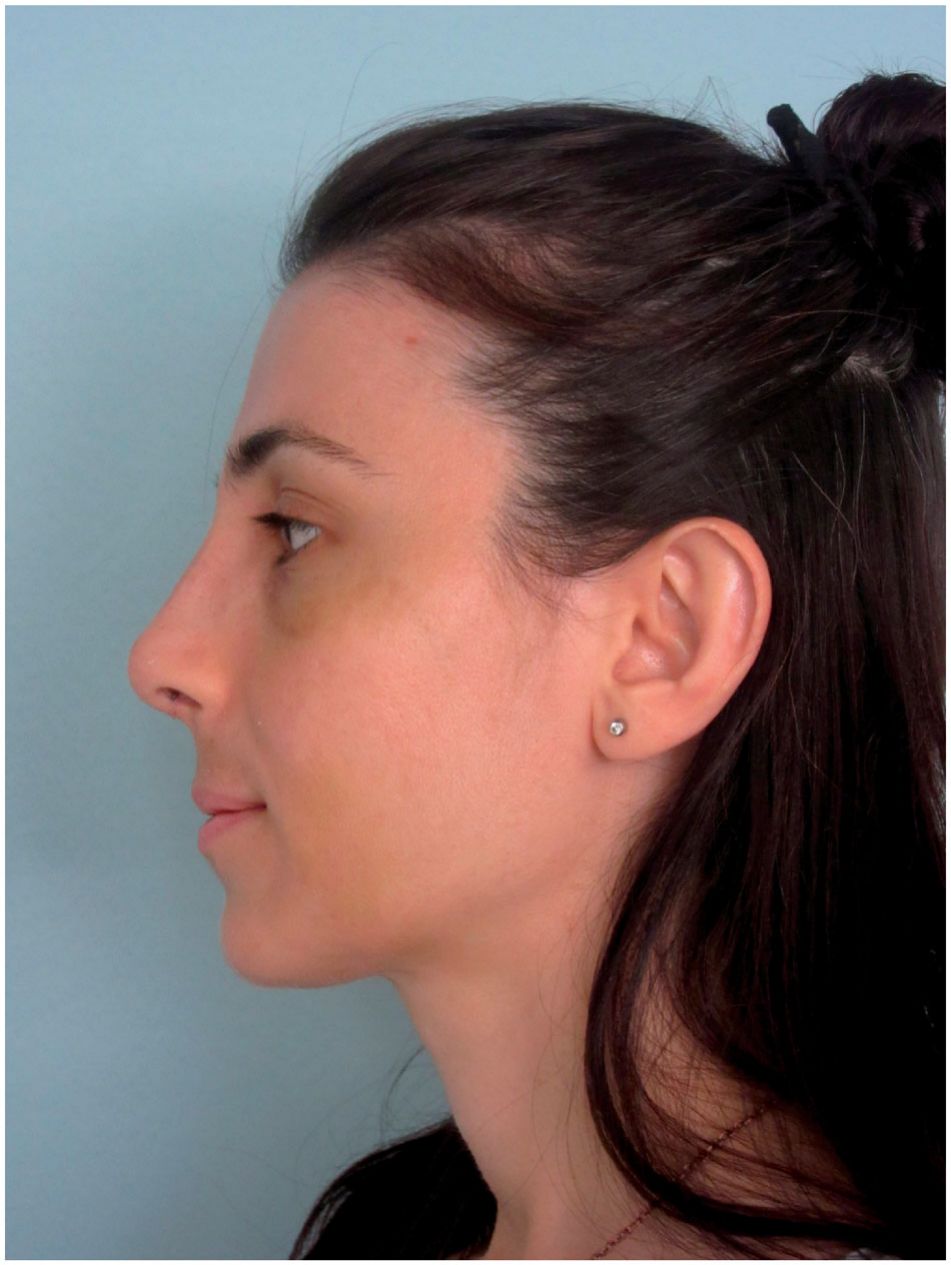
Lateral view of the patient on the 8th postoperative day.

The patient was discharged on the first postoperative day after appropriate medications were prescribed. The patient had no complaints during the controls performed on the 3rd, 5th and 8th postoperative days. The patient’s appearance on the 8th postoperative day is shown in Figures 4 and 5.

When the patient was admitted to the postoperative 1st-month control, she had no complaints and it was observed that the postoperative edema and bruising were minimal. Post-operative 3rd and 12th-month routine controls were performed.

**Figure 6.**
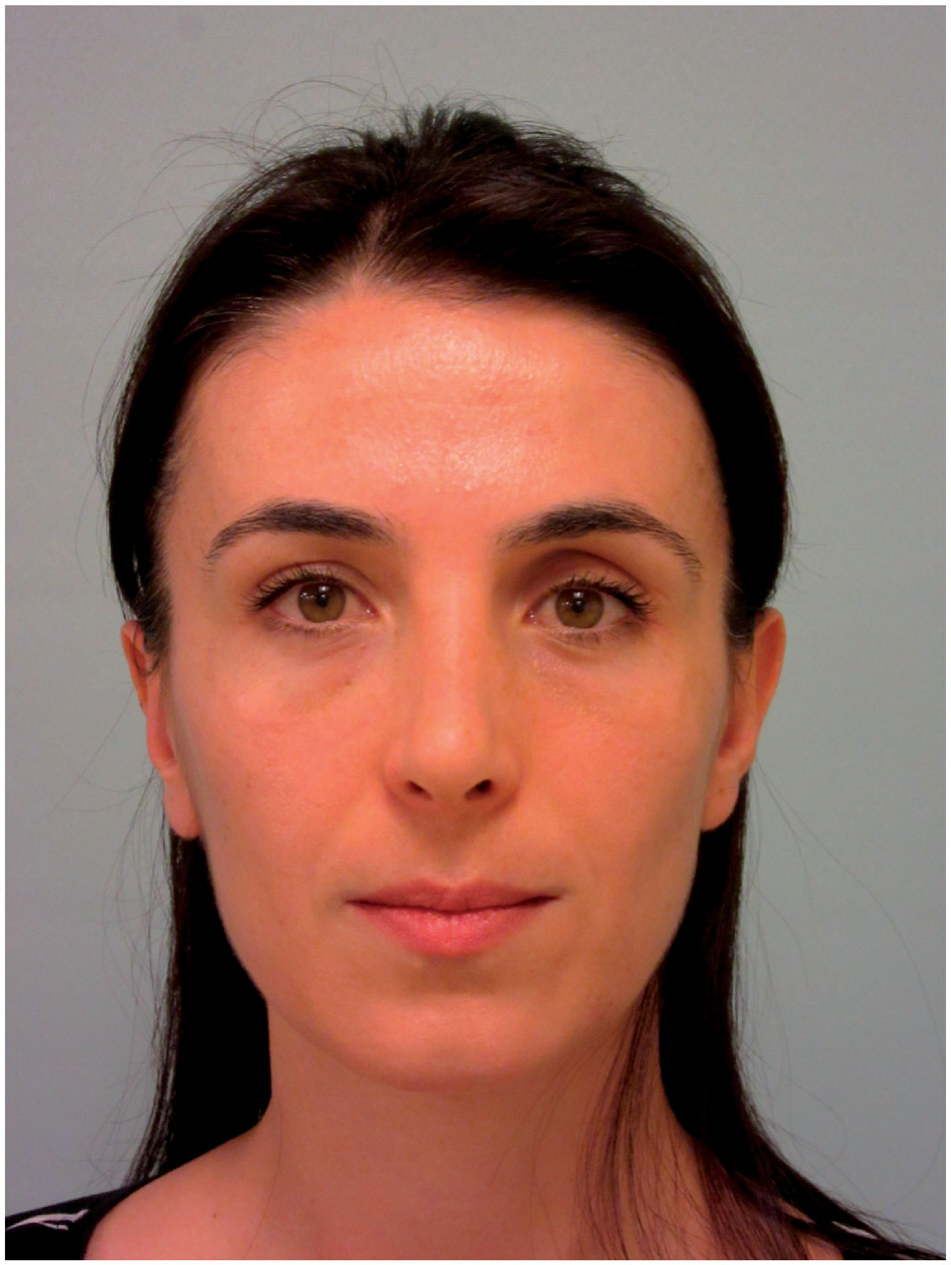
Frontal view of the patient in the 15th postoperative month.

**Figure 7.**
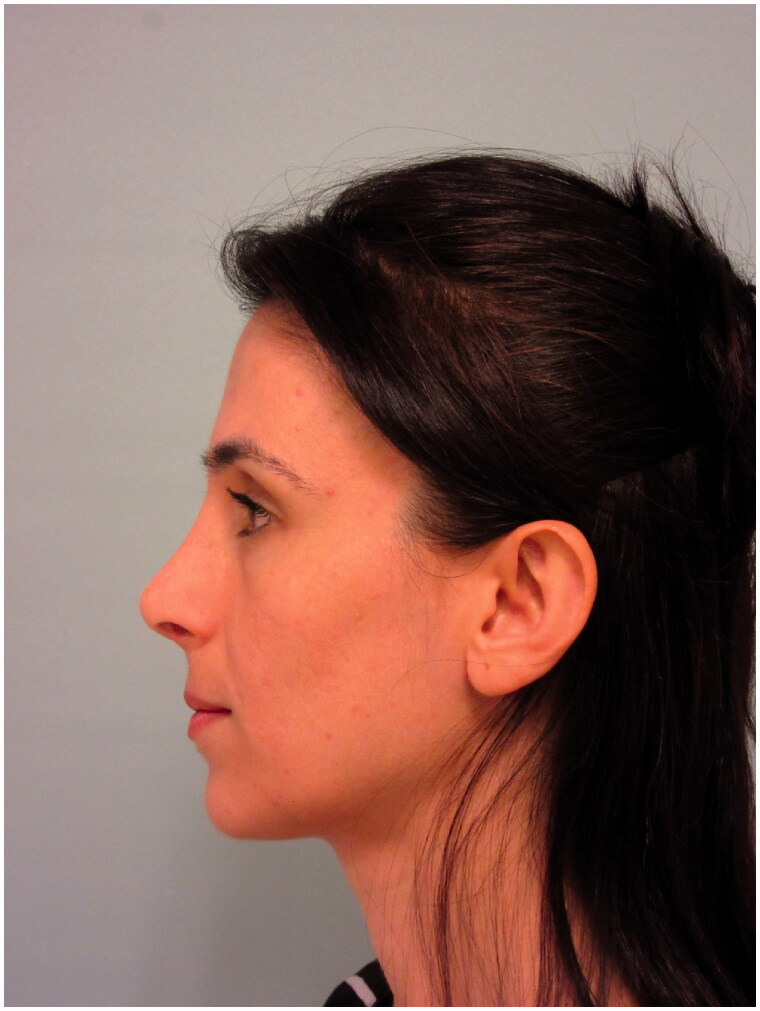
Lateral view of the patient in the 15th postoperative month.

The patient was admitted to our clinic in the postoperative 15th month with complaints of deepening of the superior sulcus in the left upper eyelid and inward collapse in the left eye. On examination of the patient, no obvious pathology was detected in eye movements and visual acuity. The patient’s appearance in the 15th postoperative month is shown in Figures 6 and 7.

**Figure 8.**
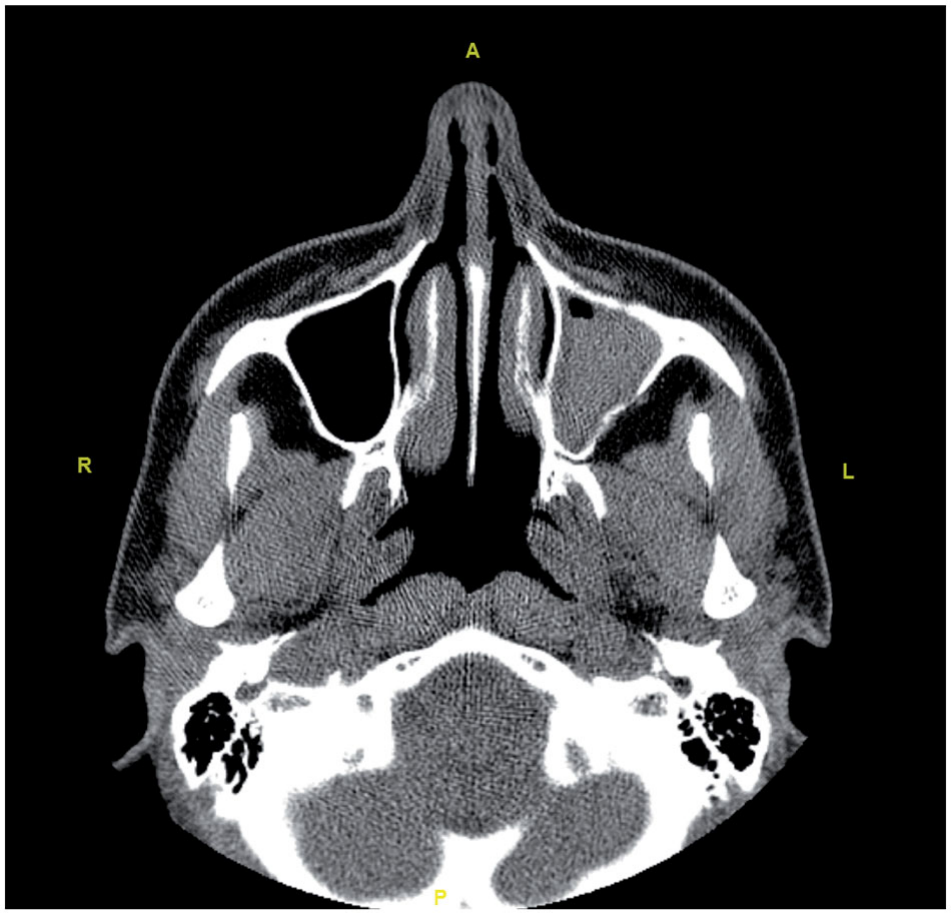
The axial section shows opacification and atelectasis of the left maxillary sinus.

**Figure 9.**
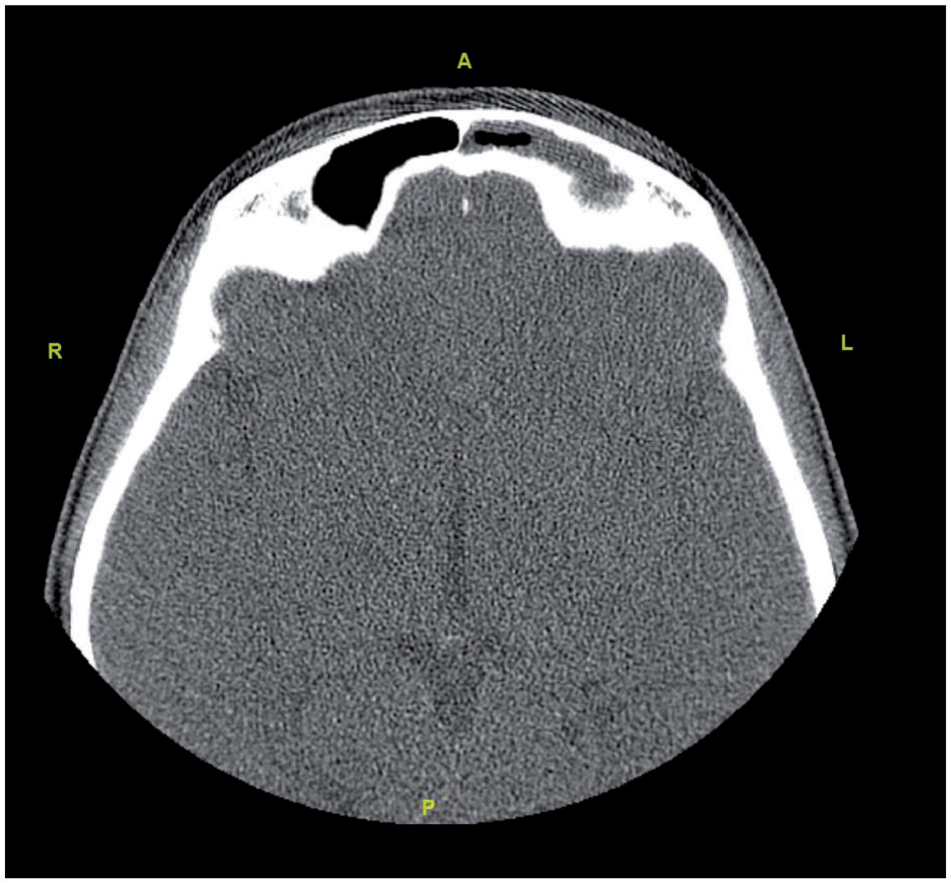
The axial section shows opacification and atelectasis in the left frontal sinus.

**Figure 10.**
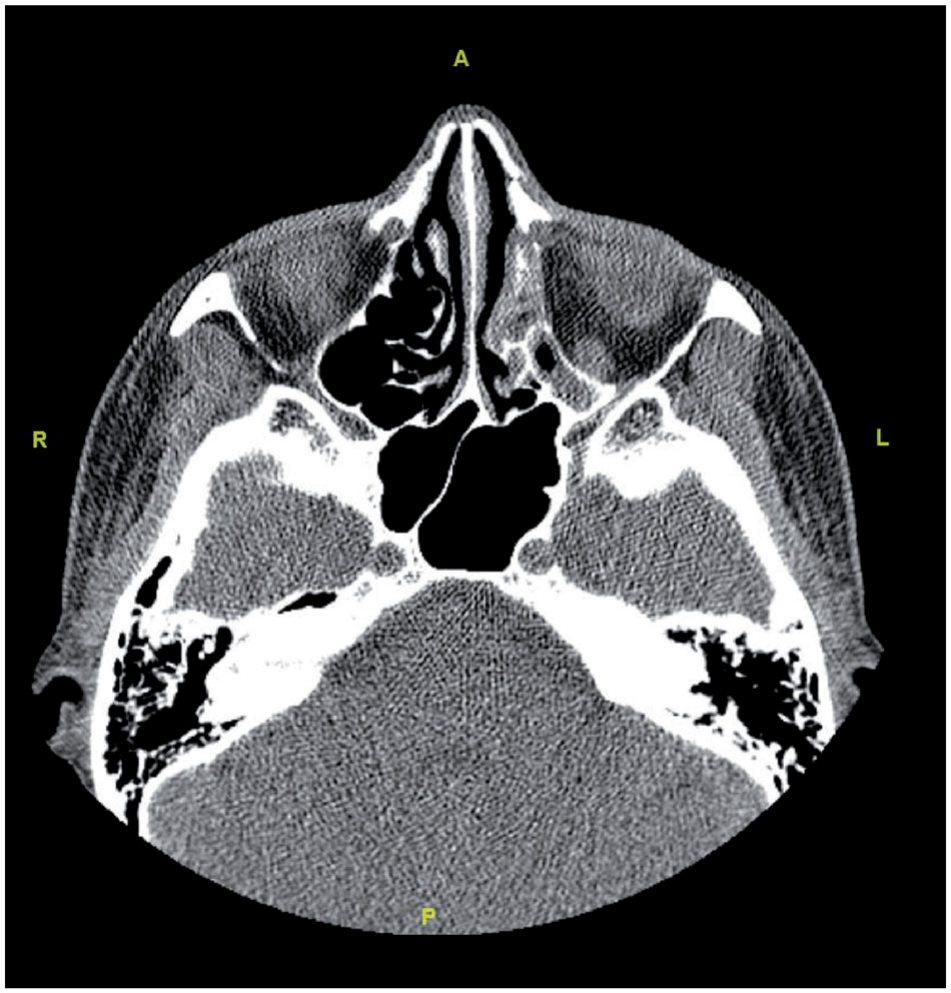
The axial section shows opacification and atelectasis in the left ethmoid sinuses.

**Figure 11.**
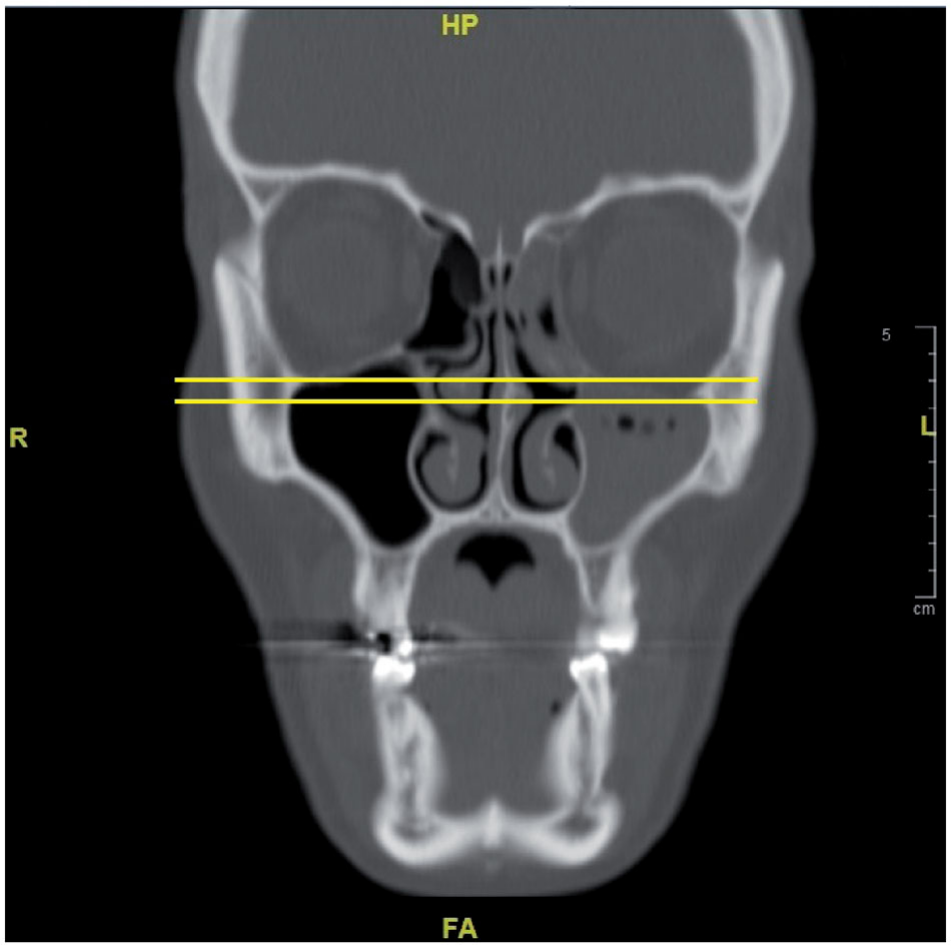
In the coronal section, opacification and atelectasis of the left maxillary sinus with collapse and downward bowing of the orbital floor can be seen.

**Figure 12.**
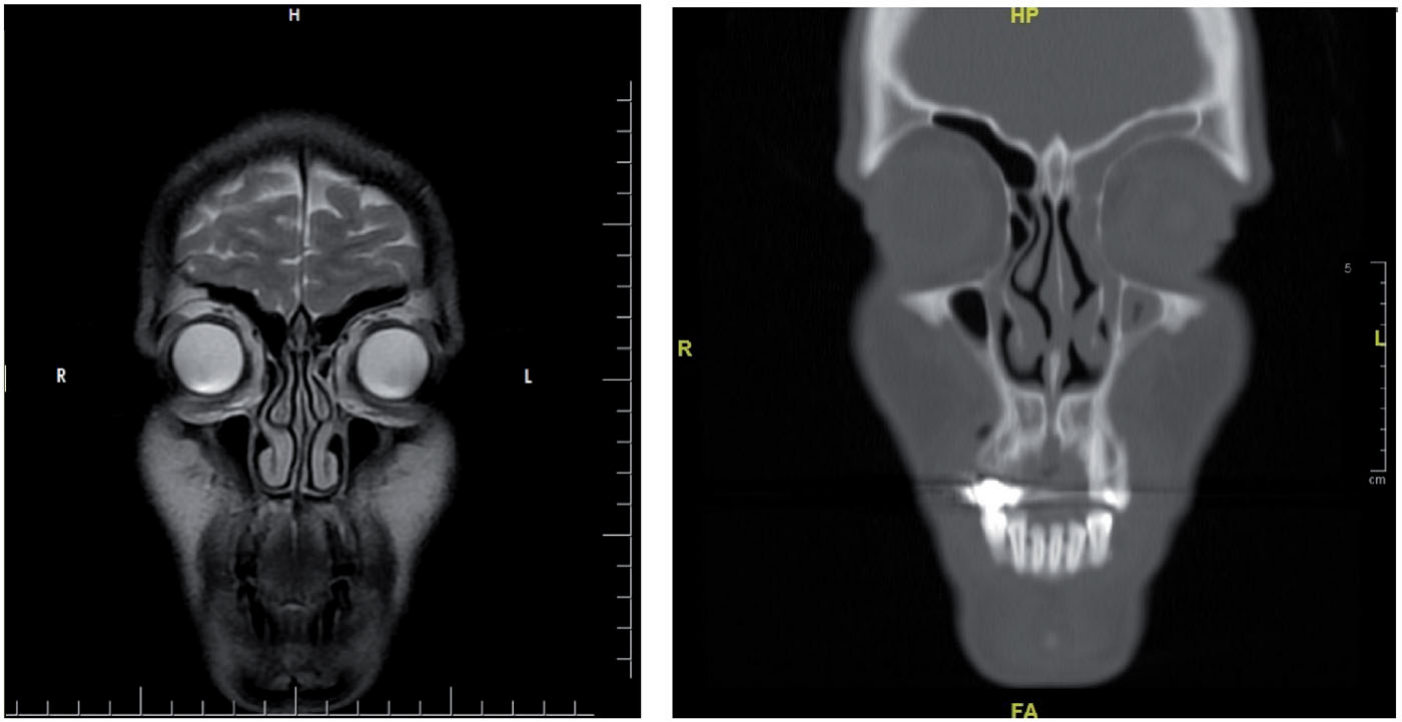
The comparison of the preoperative MR images (left) and the postoperative CT images (right). In the tomography of the patient, the opacification and atelectasis of the left maxillary, the left frontal and the left ethmoid sinuses are seen together in the coronal section. Also, the lateral displacement of the middle concha can be noted in the postoperative CT image.

**Figure 13.**
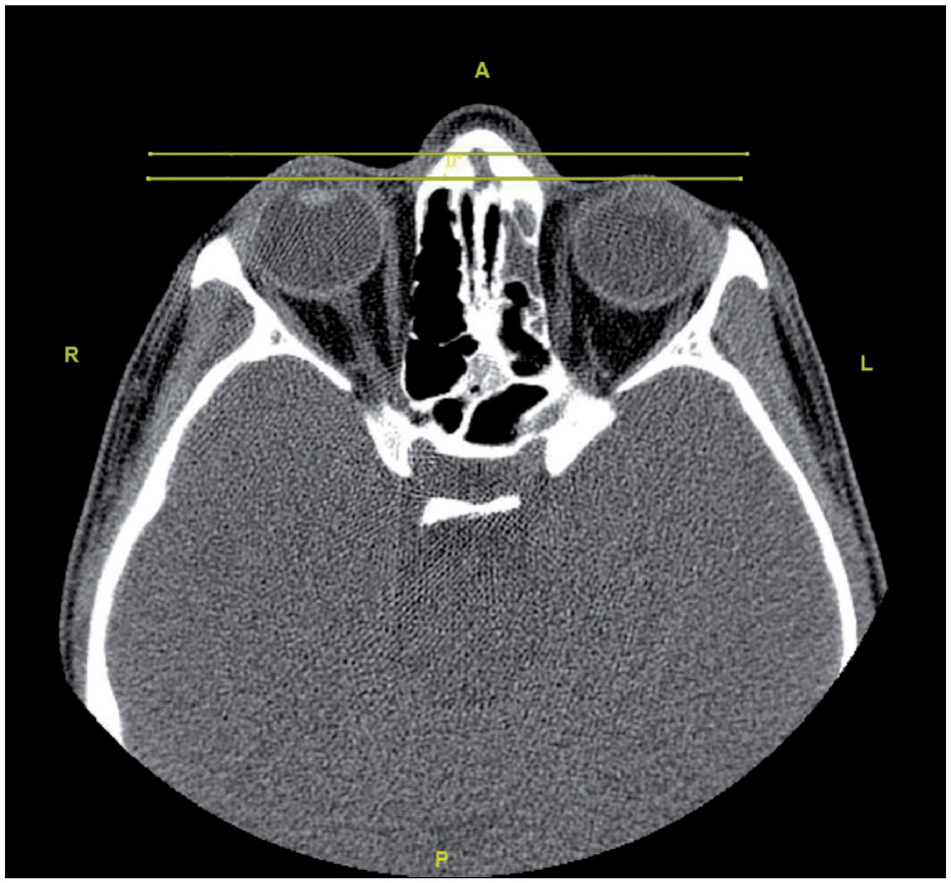
The axial section reveals that the left eyeball is located more posteriorly than the right one.

Initially, the patient was consulted to the Ophthalmology department. Hertel exophthalmometry measurement was performed by the ophthalmologists. It was 17 mm on the right and 14 mm on the left side, and the significant difference was seen between two eyes. Other ocular examinations were normal, which included ocular eye movements, fundus examination, and visual acuity. Then, the patient was reevaluated using a paranasal CT. Radiological images in all cross-sections showed that the left frontal, the left ethmoid, and the left maxillary sinuses were opacified and the left orbital floor was located more inferiorly than the right one due to atelectasis. The images of the CT scan can be seen in Figures 8–13.

With the current physical examinations and radiological imaging, the patient was diagnosed as Silent Sinus Syndrome and consulted with the ENT department. An antrostomy or antrectomy was recommended.

## Discussion

Silent Sinus Syndrome is a syndrome most commonly seen between 30-50 years of age, and is usually unilateral, with equal involvement on right and left sides and without gender predisposition. Sinus surgery, trauma or malignancies play a role in the etiology [6,7]. The patient mentioned in the case is 32 years old and there is no risk factor in etiology except the rhinoplasty operation. There is no clear information in the literature about the incidence of Silent Sinus Syndrome [8].

Although still not fully understood, the pathogenesis of the disease can be explained with three mechanisms including osteopenia and sinus wall retraction as a result of bone remodeling with Prostaglandin E_2_ mechanism in the orbital floor due to prolonged negative pressure; disruption of collagen and osteoblast replication in the orbital floor due to inflammatory cytokine release as a result of intra-sinus subclinical inflammation and congenital sinus hypoplasia, which is less favored in the majority of the literature since patients have normal sinus anatomy and function [6].

Painless orbital or facial asymmetry, deepening of the superior orbital sulcus, retraction in the upper eyelid, enophthalmos and hypoglobus without any pathology in eye movements and vision are common findings of the patients [4,9]. Malar depression and lower eyelid adipose tissue atrophy may be present in some patients [10]. Although the patient mentioned in the case had no pain, there was a posterior displacement of the eyeball and deepening of the superior sulcus.

Regarding the diagnosis, CT which is superior in bone tissue is an important imaging method for orbital and maxillary sinus pathologies [4]. Total or subtotal unilateral maxillary sinus opacification, osteomeatal unit obstruction, inferior displacement in the orbital floor, osteopenia at the orbital floor, decrease in the maxillary sinus volume, accompanying air-fluid levels and mucosal thickening are common radiological findings [4]. These findings are pathognomic for Silent Sinus Syndrome [11]. The increase in orbital volume is directly responsible for enophthalmos and hypoglobus [4]. The postoperative CT of the patient mentioned in the case addressed here was reported as: ‘Atelectasis, mucosal thickening and fluids are observed in the left frontal sinus, left ethmoid sinuses and left maxillary sinus. The left maxillary sinus volume is relatively small and its upper anterior wall and lateral wall are depressed. The lower conchae are hypertrophic. No pathology was detected in the orbital elements’.

The differential diagnosis of facial asymmetry, enophthalmos and hypoglobus includes trauma, orbital varicose veins, orbital osteomyelitis, Parry-Romberg Syndrome, Linear Scleroderma, Wegener Granulomatous, lipodystrophy, chronic sinusitis, malignant infiltration and orbital radiotherapy [4]. In chronic maxillary sinus atelectasis, which has an important place in the differential diagnosis, evident signs of inflammatory sinus disease are present [5]. Also, some other pathologies, such as alveolar maxilla trauma or perforation, dental implant application and deep root canal infection, may lead to a similar outcome. However, the patient, in this case, had no such medical history. Orbital traumas, such as orbital floor fractures, may also cause the same outcome. Brown et al. identified six patients with progressive enophthalmos due to maxillary sinus atelectasis and consequently Silent Sinus Syndrome, on average of eight months after their initial orbital floor fracture [12].

Patient history, clinical findings and radiological imaging play an important role in diagnosis. Traumatic maneuvers such as nasal speculum examination, osteotomy, inferior concha lateralization, and aggressive internal nasal splint application during rhinoplasty or septoplasty may cause osteomeatal unit obstruction, lateral movement of the middle concha towards the middle meatus, subluxation and lateral sliding of the uncinate process towards the lamina papirecea and synechia in the excretion pathway of the maxillary sinus [5].

In the case presented above, no preoperative pathology was found in the history, physical examination findings and radiological imaging. When this situation was retrospectively analyzed, it was thought that during the lateralization of the inferior concha on the left side; middle concha and the osteomeatal complex were also displaced and the drainage pathway of the maxillary, frontal, anterior and middle ethmoidal sinuses to the middle meatus were blocked.

**Figure 14.**
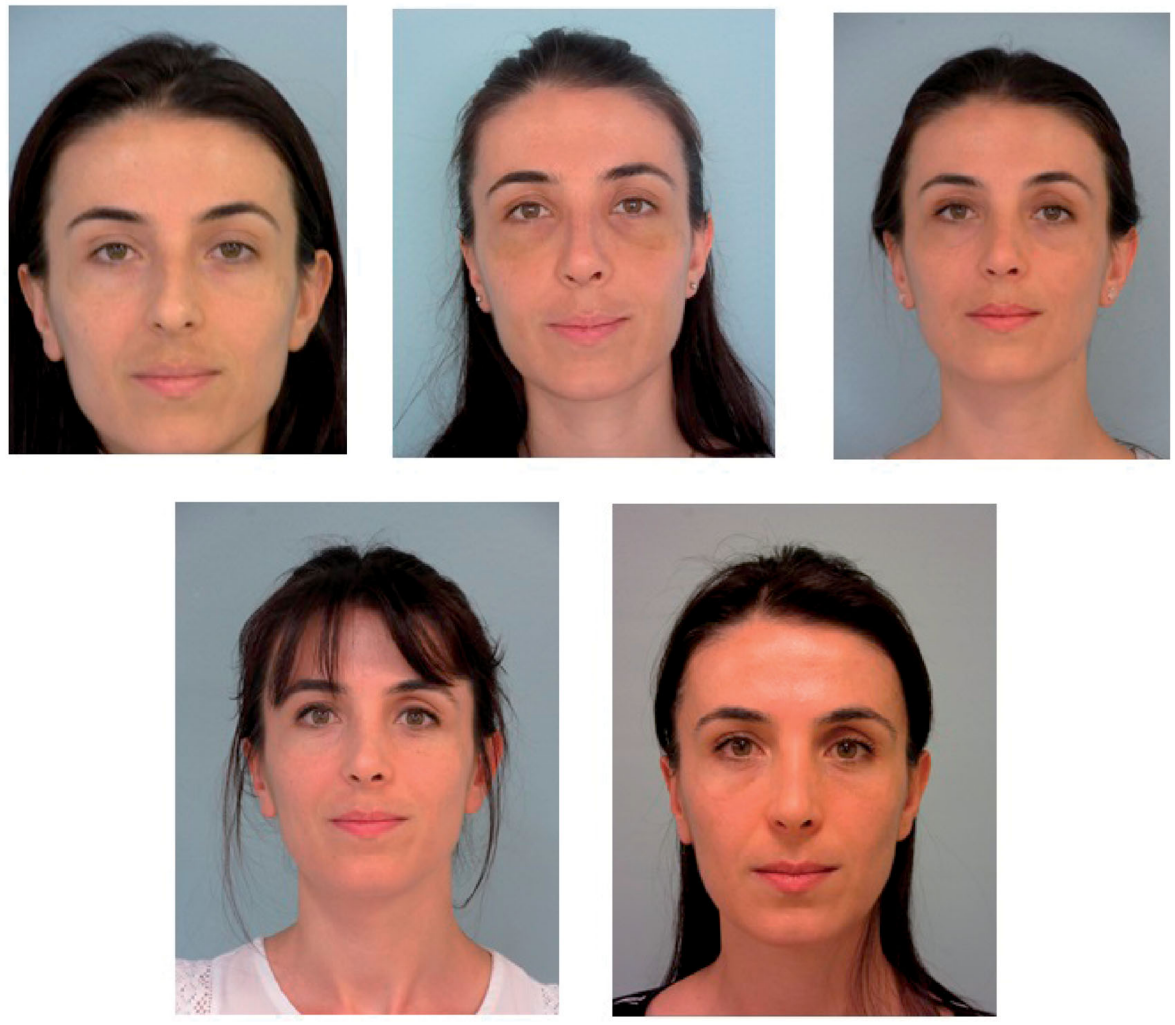
The patient’s preoperative, postoperative 8th day, postoperative 1st month, postoperative 12th month and postoperative 15th month photos are shown respectively. Pathologies of the left periorbital area which are noticed in the postoperative 1st month photo and most obvious in the postoperative 15th month photo draw attention.

When the patient’s photographs were retrospectively studied, although the patient had no complaints, it was noticed that there was a slight deepening of the superior sulcus in the postoperative 1st month. Figure 14 shows the preoperative, postoperative 8th day, postoperative 1st month, postoperative 12th month and postoperative 15th month photographs of the patient.

The treatment aims the correction of sinonasal and orbital pathologies [4]. Interventions that provide ventilation of the maxillary sinus such as endoscopic uncinectomy and maxillary sinus antrostomy constitute the sinonasal treatment. Spontaneous improvement in enophthalmos can be expected after a while after maxillary sinus decompression. Nevertheless, for aesthetic concerns, the orbital floor reconstruction can be performed using materials including titanium plaques, Medpor or autogenous tissues such as septal cartilage, iliac bone graft, split calvarial bone [4,10]. There is no consensus on whether the orbital floor reconstruction with the transconjunctival or subsilier approach will be performed concurrently with sinonasal treatment or with a staged approach [4,13]. However, if enophthalmos or diplopia is severe, simultaneous orbital floor reconstruction is preferred [14].

## Conclusion

Anatomy of the paranasal sinuses and the effects of sinuses on orbital bones and eyes are very important for plastic surgeons that often operate in the periorbital area [3]. Nasal traumas, such as concha lateralization and osteotomy, which are performed during rhinoplasty, may cause displacement of the uncinate process of the ethmoid bone and subsequently result in Silent Sinus Syndrome [5]. Therefore, in patients presenting with complications of the periorbital area after rhinoplasty, such as enophthalmos and hypoglobus, this syndrome, which is often missed or misdiagnosed, should be kept in mind in the differential diagnosis.
